# *N*-(3-Hy­droxy­phen­yl)nicotinamide

**DOI:** 10.1107/S1600536812015620

**Published:** 2012-04-21

**Authors:** Chunhua Ge, Rui Zhang, Xiangdong Zhang, Chenglong Zhang, Meiyin Zhang

**Affiliations:** a College of Chemistry, Liaoning University, Shenyang, Liaoning 110036, People’s Republic of China

## Abstract

In the title mol­ecule, C_12_H_10_N_2_O_2_, the benzene and pyridine rings form a dihedral angle of 5.01 (8)°. The amide group is twisted by 33.54 (7)° from the plane of the pyridine ring. In the crystal, mol­ecules are linked into centrosymmetric dimers *via* pairs of O—H⋯N hydrogen bonds. N—H⋯O hydrogen bonds further link dimers related into chains along the *b* axis.

## Related literature

For related structures, see: Mocilac & Gallagher (2011 ▶); Roopan *et al.* (2009 ▶). For modern aspects of boronic acid derivatives, see: Hall (2005 ▶).
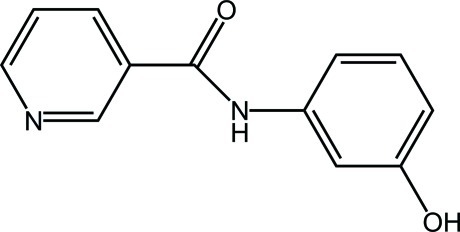


## Experimental

### 

#### Crystal data

C_12_H_10_N_2_O_2_
*M* *_r_* = 214.22
Monoclinic, 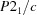

*a* = 12.1741 (13) Å
*b* = 5.2613 (6) Å
*c* = 15.3113 (16) Å
β = 94.428 (2)°
*V* = 977.79 (18) Å^3^
*Z* = 4
Mo *K*α radiation
μ = 0.10 mm^−1^
*T* = 293 K
0.35 × 0.20 × 0.18 mm

#### Data collection

Bruker SMART CCD area-detector diffractometer
Absorption correction: multi-scan (*SADABS*; Bruker, 2001 ▶) *T*_min_ = 0.952, *T*_max_ = 0.988
5813 measured reflections
1928 independent reflections
1572 reflections with *I* > 2σ(*I*)
*R*_int_ = 0.022

#### Refinement

*R*[*F* ^2^ > 2σ(*F* ^2^)] = 0.040
*wR*(*F* ^2^) = 0.098
*S* = 1.04
1928 reflections
150 parameters
H atoms treated by a mixture of independent and constrained refinement
Δρ_max_ = 0.31 e Å^−3^
Δρ_min_ = −0.17 e Å^−3^

### 

Data collection: *SMART* (Bruker, 2001 ▶); cell refinement: *SAINT* (Bruker, 2001 ▶); data reduction: *SAINT*; program(s) used to solve structure: *SHELXS97* (Sheldrick, 2008 ▶); program(s) used to refine structure: *SHELXL97* (Sheldrick, 2008 ▶); molecular graphics: *SHELXTL* (Sheldrick, 2008 ▶); software used to prepare material for publication: *SHELXL97*, *PLATON* (Spek, 2009 ▶) and *WinGX* (Farrugia, 1999 ▶).

## Supplementary Material

Crystal structure: contains datablock(s) I, global. DOI: 10.1107/S1600536812015620/cv5281sup1.cif

Structure factors: contains datablock(s) I. DOI: 10.1107/S1600536812015620/cv5281Isup2.hkl

Supplementary material file. DOI: 10.1107/S1600536812015620/cv5281Isup3.cml

Additional supplementary materials:  crystallographic information; 3D view; checkCIF report

## Figures and Tables

**Table 1:** Hydrogen-bond geometry (Å, °)

*D*—H⋯*A*	*D*—H	H⋯*A*	*D*⋯*A*	*D*—H⋯*A*
O2—H2*B*⋯N1^i^	0.82	2.00	2.817 (2)	173
N2—H2*A*⋯O1^ii^	0.83 (2)	2.29 (2)	3.107 (2)	166

Symmetry codes: (i) 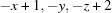; (ii) 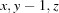.

**Fig. 1.**
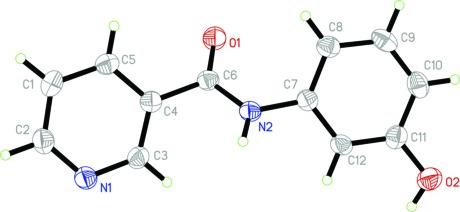
The molecular structure of the title compound with the atom-numbering scheme. Displacement ellipsoids are drawn at the 50% probability level.

## supplementary crystallographic information

### Comment

Organoboronic acid derivatives have recently received increasing interest in a broad range of biological, medicinal, and synthetic applications (Hall, 2005). Especially, such compounds have been utilized as synthetic intermediates. For example, the famous Suzuki reaction is the coupling of organoboronic acid with aryl halide. Boronic acid containing compounds employed in the cross coupling must be activated by using palladium catalyst. Other metal ion could make boronic acid in the different transformational mode.

The title compound, *N*-(3-hydroxyphenyl)nicotinamide, is obtained by reaction of *N*-(3-phenylboronic acid)nicotinamide with copper(II) ion. The molecular structure is shown in Fig. 1. Conformational studies show that substituent of the phenyl ring is one of key factors for solid state molecular conformations and supramolecular aggregation. Comparisons between *N*-phenylnicotinamide and *N*-(3-hydroxyphenyl)nicotinamide reveal that the dihedral angle between the phenyl and pyridine rings is 64.81 (1) ° (Roopan *et al.*, 2009) in the former and 5.02 (8) ° in the latter. This value in *N*-(3-methylphenyl)nicotinamide is 57.23 (6) ° (Mocilac & Gallagher, 2011). Oxygen atom from amide group and nitrogen atom from pyridine ring in *N*-phenylnicotinamide are on the same side of the molecule. The distribution of corresponding atoms in *N*-(3-methylphenyl)nicotinamide is similar to that of *N*-phenylnicotinamide, but contrary to that of *N*-(3-hydroxyphenyl)nicotinamide.

In the crystal structure, the molecules are paired into centrosymmetric dimers *via* O—H···N hydrogen bonds (Table 1). Intermolecular N—H···O hydrogen bonds (Table 1) link further these dimers related be translation along axis *b* into chains.

### Experimental

*N*-(3-Phenylboronic acid)nicotinamide (10 mmol) was added to 20 ml e thanol-water(v:v=8:2), followed by the dropwise addition of copper nitrate(5 mmol) in 5 ml water. The mixture was stirred at room temperature for 8 h. After filtered, the filtrate was evaporated. Crystals were obtained after about two weeks.

### Refinement

The amide H atom was located in Fourier different map and refined isotropically. All other H atoms were placed in geometrically idealized positions (C*sp*^2^—H = 0.93, and O—H = 0.82) and refined as riding, with *U*_iso_(H) = 1.2*U*_eq_(C) and *U*_iso_(H) = 1.5*U*_eq_(O).

### Crystal data

**Table:**

C_12_H_10_N_2_O_2_	*F*(000) = 448
*M**_r_* = 214.22	*D*_x_ = 1.455 Mg m^−^^3^
Monoclinic, *P*2_1_/*c*	Mo *K*α radiation, λ = 0.71073 Å
Hall symbol: -P 2ybc	Cell parameters from 82 reflections
*a* = 12.1741 (13) Å	θ = 2.2–23.3°
*b* = 5.2613 (6) Å	µ = 0.10 mm^−^^1^
*c* = 15.3113 (16) Å	*T* = 293 K
β = 94.428 (2)°	Block, colourless
*V* = 977.79 (18) Å^3^	0.35 × 0.20 × 0.18 mm
*Z* = 4	

### Data collection

**Table:**

Bruker SMART CCD area-detector diffractometer	1928 independent reflections
Radiation source: fine-focus sealed tube	1572 reflections with *I* > 2σ(*I*)
Graphite monochromator	*R*_int_ = 0.022
φ and ω scans	θ_max_ = 26.1°, θ_min_ = 1.7°
Absorption correction: multi-scan (*SADABS*; Bruker, 2001)	*h* = −15→15
*T*_min_ = 0.952, *T*_max_ = 0.988	*k* = −6→4
5813 measured reflections	*l* = −18→18

### Refinement

**Table:**

Refinement on *F*^2^	Primary atom site location: structure-invariant direct methods
Least-squares matrix: full	Secondary atom site location: difference Fourier map
*R*[*F*^2^ > 2σ(*F*^2^)] = 0.040	Hydrogen site location: inferred from neighbouring sites
*wR*(*F*^2^) = 0.098	H atoms treated by a mixture of independent and constrained refinement
*S* = 1.04	*w* = 1/[σ^2^(*F*_o_^2^) + (0.0441*P*)^2^ + 0.3178*P*] where *P* = (*F*_o_^2^ + 2*F*_c_^2^)/3
1928 reflections	(Δ/σ)_max_ < 0.001
150 parameters	Δρ_max_ = 0.31 e Å^−^^3^
0 restraints	Δρ_min_ = −0.17 e Å^−^^3^

### Special details

**Table:**

Geometry. All e.s.d.'s (except the e.s.d. in the dihedral angle between two l.s. planes) are estimated using the full covariance matrix. The cell e.s.d.'s are taken into account individually in the estimation of e.s.d.'s in distances, angles and torsion angles; correlations between e.s.d.'s in cell parameters are only used when they are defined by crystal symmetry. An approximate (isotropic) treatment of cell e.s.d.'s is used for estimating e.s.d.'s involving l.s. planes.
Refinement. Refinement of *F*^2^ against ALL reflections. The weighted *R*-factor *wR* and goodness of fit *S* are based on *F*^2^, conventional *R*-factors *R* are based on *F*, with *F* set to zero for negative *F*^2^. The threshold expression of *F*^2^ > σ(*F*^2^) is used only for calculating *R*-factors(gt) *etc*. and is not relevant to the choice of reflections for refinement. *R*-factors based on *F*^2^ are statistically about twice as large as those based on *F*, and *R*- factors based on ALL data will be even larger.

### Fractional atomic coordinates and isotropic or equivalent isotropic displacement parameters (Å^2^)

**Table:**

	*x*	*y*	*z*	*U*_iso_*/*U*_eq_	
O2	0.10112 (9)	0.0774 (2)	0.89614 (8)	0.0393 (3)	
H2B	0.1369	−0.0179	0.9298	0.059*	
N2	0.46607 (10)	0.4089 (3)	0.86950 (8)	0.0293 (3)	
C12	0.28388 (12)	0.2438 (3)	0.88031 (10)	0.0289 (3)	
H12	0.3155	0.1113	0.9137	0.035*	
O1	0.51225 (9)	0.8283 (2)	0.87911 (8)	0.0403 (3)	
C5	0.73908 (12)	0.6822 (3)	0.88082 (10)	0.0309 (4)	
H5	0.7221	0.8299	0.8493	0.037*	
N1	0.79027 (10)	0.2474 (3)	0.97897 (9)	0.0327 (3)	
C11	0.17038 (12)	0.2524 (3)	0.86264 (10)	0.0310 (4)	
C6	0.53822 (12)	0.6020 (3)	0.88392 (10)	0.0279 (3)	
C2	0.86888 (13)	0.3961 (3)	0.95025 (10)	0.0339 (4)	
H2	0.9420	0.3493	0.9632	0.041*	
C9	0.19122 (13)	0.6308 (3)	0.77876 (10)	0.0354 (4)	
H9	0.1596	0.7605	0.7441	0.042*	
C8	0.30484 (13)	0.6289 (3)	0.79759 (10)	0.0310 (4)	
H8	0.3490	0.7560	0.7767	0.037*	
C4	0.65565 (12)	0.5257 (3)	0.90684 (9)	0.0267 (3)	
C7	0.35048 (12)	0.4310 (3)	0.84863 (9)	0.0267 (3)	
C1	0.84752 (13)	0.6154 (3)	0.90241 (11)	0.0349 (4)	
H1	0.9048	0.7159	0.8851	0.042*	
C3	0.68548 (12)	0.3122 (3)	0.95639 (10)	0.0307 (4)	
H3	0.6298	0.2087	0.9749	0.037*	
C10	0.12390 (13)	0.4454 (3)	0.81013 (10)	0.0349 (4)	
H10	0.0482	0.4501	0.7962	0.042*	
H2A	0.4898 (13)	0.261 (4)	0.8741 (10)	0.030 (5)*	

### Atomic displacement parameters (Å^2^)

**Table:**

	*U*^11^	*U*^22^	*U*^33^	*U*^12^	*U*^13^	*U*^23^
O2	0.0341 (6)	0.0315 (7)	0.0528 (8)	0.0008 (5)	0.0074 (5)	0.0036 (5)
N2	0.0292 (7)	0.0198 (7)	0.0385 (8)	0.0021 (6)	−0.0012 (5)	0.0000 (6)
C12	0.0318 (8)	0.0221 (8)	0.0323 (8)	0.0038 (6)	−0.0013 (6)	−0.0014 (6)
O1	0.0347 (6)	0.0231 (6)	0.0624 (8)	0.0011 (5)	−0.0004 (5)	−0.0003 (5)
C5	0.0350 (8)	0.0266 (8)	0.0311 (8)	−0.0025 (7)	0.0027 (6)	0.0010 (6)
N1	0.0306 (7)	0.0273 (7)	0.0396 (7)	0.0001 (6)	−0.0012 (6)	−0.0012 (6)
C11	0.0303 (8)	0.0270 (8)	0.0360 (8)	−0.0001 (7)	0.0037 (6)	−0.0074 (7)
C6	0.0301 (8)	0.0238 (8)	0.0298 (8)	0.0004 (6)	0.0027 (6)	−0.0003 (6)
C2	0.0276 (8)	0.0333 (9)	0.0402 (9)	0.0008 (7)	−0.0006 (7)	−0.0051 (7)
C9	0.0422 (9)	0.0320 (9)	0.0311 (8)	0.0121 (8)	−0.0025 (7)	−0.0001 (7)
C8	0.0366 (8)	0.0268 (8)	0.0294 (8)	0.0010 (7)	0.0007 (6)	−0.0011 (6)
C4	0.0299 (8)	0.0228 (8)	0.0274 (7)	0.0000 (6)	0.0023 (6)	−0.0049 (6)
C7	0.0283 (7)	0.0229 (8)	0.0284 (7)	0.0021 (6)	−0.0002 (6)	−0.0044 (6)
C1	0.0301 (8)	0.0372 (9)	0.0381 (9)	−0.0065 (7)	0.0062 (7)	−0.0026 (7)
C3	0.0295 (8)	0.0266 (8)	0.0360 (8)	−0.0034 (6)	0.0021 (6)	−0.0018 (7)
C10	0.0289 (8)	0.0383 (10)	0.0370 (9)	0.0070 (7)	−0.0010 (7)	−0.0069 (7)

### Geometric parameters (Å, º)

**Table:**

O2—C11	1.3741 (19)	C11—C10	1.388 (2)
O2—H2B	0.8200	C6—C4	1.500 (2)
N2—C6	1.350 (2)	C2—C1	1.381 (2)
N2—C7	1.4234 (19)	C2—H2	0.9300
N2—H2A	0.831 (18)	C9—C10	1.384 (2)
C12—C7	1.387 (2)	C9—C8	1.391 (2)
C12—C11	1.388 (2)	C9—H9	0.9300
C12—H12	0.9300	C8—C7	1.391 (2)
O1—C6	1.2325 (18)	C8—H8	0.9300
C5—C1	1.381 (2)	C4—C3	1.388 (2)
C5—C4	1.389 (2)	C1—H1	0.9300
C5—H5	0.9300	C3—H3	0.9300
N1—C2	1.336 (2)	C10—H10	0.9300
N1—C3	1.3396 (19)		
			
C11—O2—H2B	109.5	C10—C9—H9	119.0
C6—N2—C7	126.48 (14)	C8—C9—H9	119.0
C6—N2—H2A	118.2 (12)	C9—C8—C7	117.99 (15)
C7—N2—H2A	115.3 (11)	C9—C8—H8	121.0
C7—C12—C11	120.56 (14)	C7—C8—H8	121.0
C7—C12—H12	119.7	C3—C4—C5	118.02 (14)
C11—C12—H12	119.7	C3—C4—C6	123.28 (13)
C1—C5—C4	119.16 (15)	C5—C4—C6	118.62 (14)
C1—C5—H5	120.4	C12—C7—C8	120.59 (14)
C4—C5—H5	120.4	C12—C7—N2	117.24 (13)
C2—N1—C3	117.27 (14)	C8—C7—N2	122.16 (14)
O2—C11—C12	122.38 (14)	C2—C1—C5	118.47 (15)
O2—C11—C10	118.15 (14)	C2—C1—H1	120.8
C12—C11—C10	119.47 (14)	C5—C1—H1	120.8
O1—C6—N2	123.82 (14)	N1—C3—C4	123.40 (14)
O1—C6—C4	120.52 (14)	N1—C3—H3	118.3
N2—C6—C4	115.67 (13)	C4—C3—H3	118.3
N1—C2—C1	123.60 (15)	C9—C10—C11	119.38 (14)
N1—C2—H2	118.2	C9—C10—H10	120.3
C1—C2—H2	118.2	C11—C10—H10	120.3
C10—C9—C8	121.96 (15)		

### Hydrogen-bond geometry (Å, º)

**Table:**

*D*—H···*A*	*D*—H	H···*A*	*D*···*A*	*D*—H···*A*
O2—H2*B*···N1^i^	0.82	2.00	2.817 (2)	173
N2—H2*A*···O1^ii^	0.83 (2)	2.29 (2)	3.107 (2)	166

Symmetry codes: (i) −*x*+1, −*y*, −*z*+2; (ii) *x*, *y*−1, *z*.
